# The distribution and degradation of radiolabeled superparamagnetic iron oxide nanoparticles and quantum dots in mice

**DOI:** 10.3762/bjnano.6.11

**Published:** 2015-01-09

**Authors:** Denise Bargheer, Artur Giemsa, Barbara Freund, Markus Heine, Christian Waurisch, Gordon M Stachowski, Stephen G Hickey, Alexander Eychmüller, Jörg Heeren, Peter Nielsen

**Affiliations:** 1 Department of Biochemistry and Molecular Cell Biology, University Medical Center Hamburg-Eppendorf, Martinistr. 52, 20246 Hamburg, Germany; 2 Institute of Physical Chemistry and Electrochemistry, Technical University of Dresden, Bergstr. 66b, 01069 Dresden, Germany

**Keywords:** biodistribution, chromium(III), ^51^Cr, quantum dots, SPIOs, zinc metabolism, ^65^Zn

## Abstract

^51^Cr-labeled, superparamagnetic, iron oxide nanoparticles (^51^Cr-SPIOs) and ^65^Zn-labeled CdSe/CdS/ZnS-quantum dots (^65^Zn-Qdots) were prepared using an easy, on demand, exchange-labeling technique and their particokinetic parameters were studied in mice after intravenous injection. The results indicate that the application of these heterologous isotopes can be used to successfully mark the nanoparticles during initial distribution and organ uptake, although the ^65^Zn-label appeared not to be fully stable. As the degradation of the nanoparticles takes place, the individual transport mechanisms for the different isotopes must be carefully taken into account. Although this variation in transport paths can bring new insights with regard to the respective trace element homeostasis, it can also limit the relevance of such trace material-based approaches in nanobioscience. By monitoring ^51^Cr-SPIOs after oral gavage, the gastrointestinal non-absorption of intact SPIOs in a hydrophilic or lipophilic surrounding was measured in mice with such high sensitivity for the first time. After intravenous injection, polymer-coated, ^65^Zn-Qdots were mainly taken up by the liver and spleen, which was different from that of ionic ^65^ZnCl_2._ Following the label for 4 weeks, an indication of substantial degradation of the nanoparticles and the release of the label into the Zn pool was observed. Confocal microscopy of rat liver cryosections (prepared 2 h after intravenous injection of polymer-coated Qdots) revealed a colocalization with markers for Kupffer cells and liver sinusoidal endothelial cells (LSEC), but not with hepatocytes. In J774 macrophages, fluorescent Qdots were found colocalized with lysosomal markers. After 24 h, no signs of degradation could be detected. However, after 12 weeks, no fluorescent nanoparticles could be detected in the liver cryosections, which would confirm our ^65^Zn data showing a substantial degradation of the polymer-coated CdSe/CdS/ZnS-Qdots in the liver.

## Introduction

Quantum dots (Qdots) are semiconductor nanocrystals (2–100 nm in diameter) that combine a strong, size-tunable photoluminescence with robust photostability, which makes them a highly promising tool for various applications in nanobioscience and nanomedicine [1–6].

Since many Qdots contain cadmium or other toxic elements, the release of potential toxic metals is a major concern with respect to biosafety. Many studies have demonstrated the toxicity of various Qdots in cell culture [7–12]. However, when dosing was applied under physiological conditions in mice and rats, no abnormal behavior or tissue damage was observed over the period of months after systemic administration of Qdots [13–15]. Therefore, the relevancy of the in vitro data have been called into question regarding the application in animals or even for future application in humans [15–16]. The organ-specific dose for in vivo experiments may not be high enough to induce detectable acute toxicity. However, when Qdots are retained by animals, long-term toxicity may be a problem. As a consequence, the potential harmful effects of Qdots in vivo remain unclear thus far, leaving many open questions. Future toxicity studies should be more standardized and systematic, because the methodological variability in the available literature to date makes it difficult to compare and contrast results.

One important aspect in nanotoxicity evaluation is reliable information on the distribution and metabolism for each individual Qdot in vivo. To date, there exists only a limited number of comprehensive studies concerning in vivo behavior of Qdots [17–19]. One reason is the lack of appropriate techniques to reliably quantify the dynamic variation of Qdots in living animals. Fluorescent imaging has low spatial resolution and limited penetration depth, and quantification based only on such methods is limited.

Functionalized SPIOs are also interesting candidates for nanomedical applications because of their magnetic properties that allow specific targeting of early tumor or arteriosclerotic lesions, which can be closely monitored by magnetic resonance imaging (MRI). In contrast to Qdots, iron-based nanoparticles are known to be less toxic given that iron is an essential trace element and the iron transport pathways and the regulation of iron homeostasis are quite well understood [20–21]. However, similar to QDs, the fate of injected SPIOs is also still not completely understood. The key problem again is the lack of a reliable quantitative technique for in vivo studies. In MRI, the correlation of the relaxation times to the local nanoparticle concentrations is difficult due to possible agglomeration, where the increase of hydrodynamic diameters caused by opsonization and the difficulty in the quantification of the degradation and the cellular uptake of particles [22–23]. In addition, most tissues contain substantial concentration of background iron that can be higher than the amount of injected nanoparticulate iron.

For use in animal studies and for the eventual transfer to clinical applications, more detailed information on the biocompatibility, in vivo kinetics, targeting efficacy and the acute as well as the chronic toxicity of both nanoparticle systems is needed. We are interested in techniques that allow the quantification of nanoparticles in vivo and have already developed a post-synthetic method to radiolabel the cores of superparamagnetic iron oxide particles (SPIOs) [24]. In this sense, radiolabeling could become a powerful tool for the full quantification of the particokinetic details. However, it requires special equipment and knowledge, and the selection of appropriate isotopes is critical. We here report on the advantages and disadvantages of labeling SPIOs and Qdots with heterologous radionuclides (^65^Zn for Cd, ^51^Cr for Fe) for the study of the distribution and degradation of NPs in vivo.

## Results and Discussion

### Radiolabeling of ^51^Cr-SPIOs and ^65^Zn-Qdots

**Figure 1:**
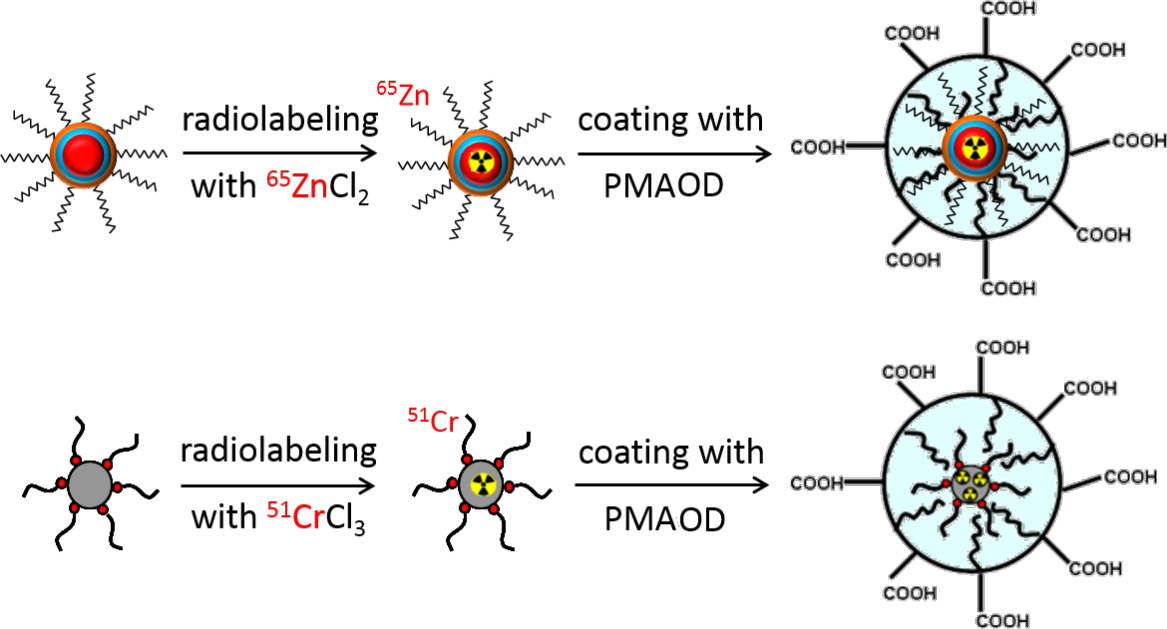
Postsynthetic labeling of quantum dots or SPIOs by incubation of monodisperse, oleic acid-stabilized core particles (CdSe/CdS/ZnS-quantum dots, 5.5 or 7 nm; SPIOs, 11 nm) in chloroform with water-free ^51^CrCl_3_ or ^65^ZnCl_2_. The hydrophobic cores were then transferred into aqueous medium by encapsulation with a well-characterized amphiphilic polymer, poly[maleic anhydride-*alt*-(1-octadecene)] (PMAOD).

The cores of SPIOs and Qdots were radiolabeled with different γ-emitting isotopes and basic parameters regarding their biodistribution and degradation were studied. It was previously shown that oleic acid-stabilized, hydrophobic, monodisperse, iron oxide cores can easily incorporate water-free ^59^FeCl_3_ [24]. This results in the stable labeling of the core and allows a quasi “on-demand” synthesis of ^59^Fe-SPIOs designed for in vivo experiments in animal models. It was coincidentally found that the SPIOs could similarly be tagged with ^51^CrCl_3_, likely due to the similarity between the Fe(III)- and Cr(III)-oxide chemistry. An attempted incorporation of the divalent cation ^65^ZnCl_2_ in the iron oxide core of SPIOs under similar experimental conditions was unsuccessful. However, a distinct incorporation of ^65^ZnCl_2_ occurred when CdSe/CdS/ZnS core/shell/shell quantum dots were synthesized (Figure 1).

Both hydrophobic nanoparticle cores were encapsulated using the same polymer to render them water soluble. This resulted in similar nanoparticles (comparable size, surface chemistry and charge), despite the completely different core material. This was proven when the biodistribution was compared using fluorescent Qdots and intravital microscopy in mice or MRI measurements in mice and TEM in ex vivo samples for SPIOs (data not shown). However, it should be noted that this type of radiolabeling with nonidentical radionuclides (^51^Cr for Fe, ^65^Zn for Cd) raises questions on the validity of the label data along the transport and the degradation pathways of the particles.

### ^51^Cr-labeling of SPIOs

**Figure 2:**
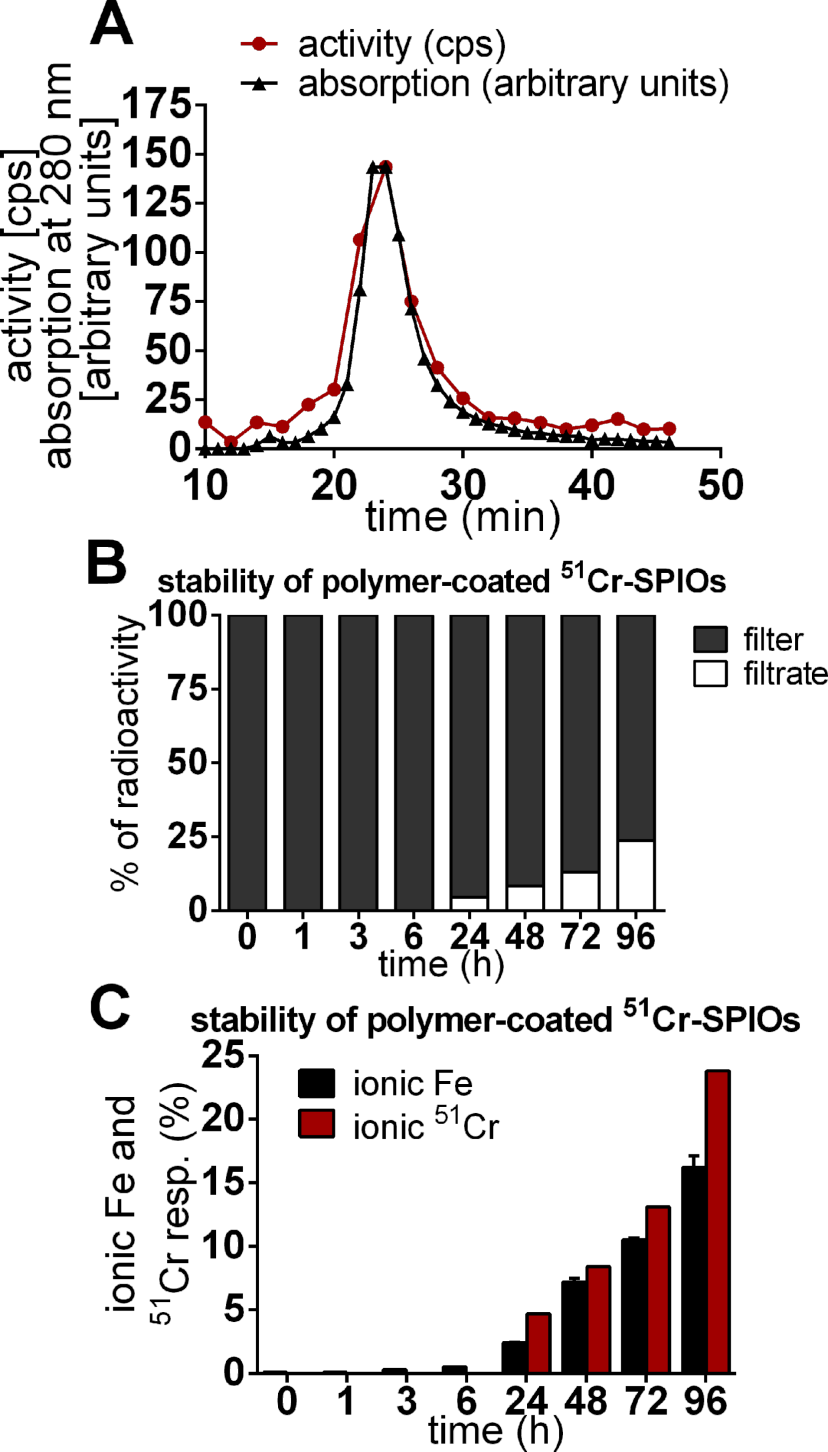
Stability of ^51^Cr-radiolabeled SPIOs with a polymer shell. (A) Size-exclusion chromatography (SEC) results of the polymer-coated ^51^Cr-SPIOs. The radioactivity measured in fractions corresponds to the elution of the NPs measured by UV absorption at 280 nm. (B) Release of ^51^Cr^3+^ from radiolabeled nanoparticles when incubated with 0.1 M HCl at room temperature. A centrifuge was used for the separation of released chromium ions (filtrate) from nanoparticles (filter) at distinct times during the degradation of the particles. (C) Release of Fe^3+^ and ^51^Cr^3+^ from degraded iron oxide cores was measured in the filtrate via atomic absorption spectrometry and ^51^Cr-γ-counting. The results indicate the constant release of iron and chromium over time during the degradation of the particles.

The exchange labeling with ^51^CrCl_3_ resulted in a stable and homogenous labeling of the iron oxide core in the model SPIOs as shown by fast protein liquid chromatography, filtration experiments and the acid-catalyzed release of iron and ^51^Cr (Figure 2).

**Figure 3:**
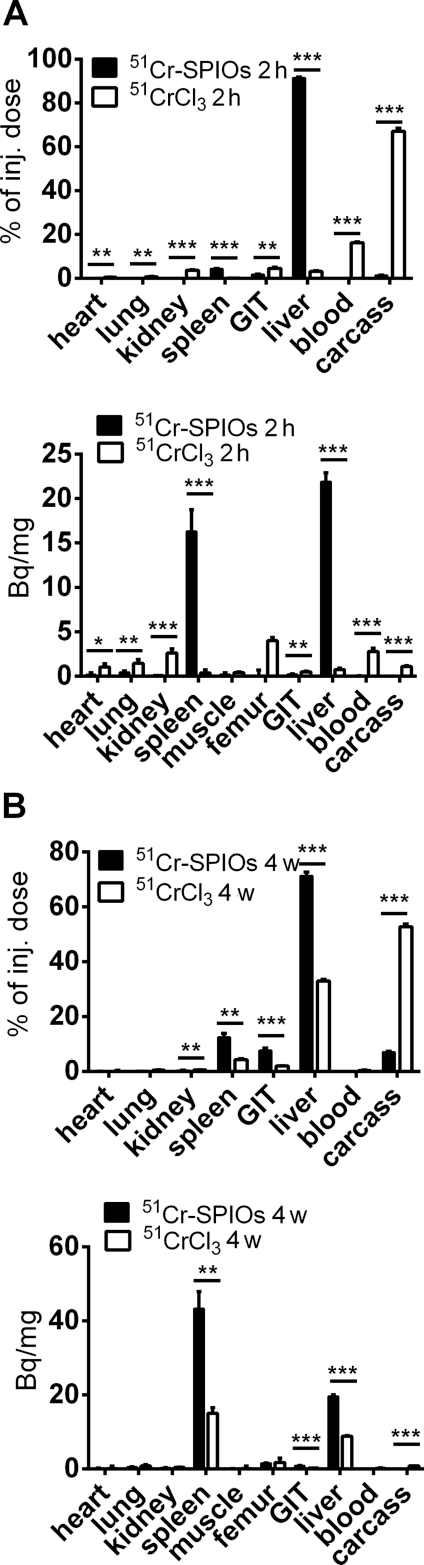
Distribution and degradation of ^51^Cr-SPIOs in comparison with ^51^CrCl_3_ after intravenous injection in groups of mice (*n* = 4). (A) ^51^Cr activity in organs and tissue 2 h and (B) 4 weeks (4 w) after injection. Data are presented as mean values ± standard error of the mean. Asterisks indicate significant differences (*p* < 0.05).

When injected intravenously into mice, the ^51^Cr-labeled SPIOs were taken up by the liver and spleen almost completely as seen by the organ distribution results (Figure 3) two hours after administration. The distribution of a trace dose of ^51^CrCl_3_ is more pronounced in the blood and carcass and was overall very different from the ^51^Cr-SPIOs. The short time distribution is in good agreement with earlier results in rats, including also a transient storage in bone [25].

**Figure 4:**
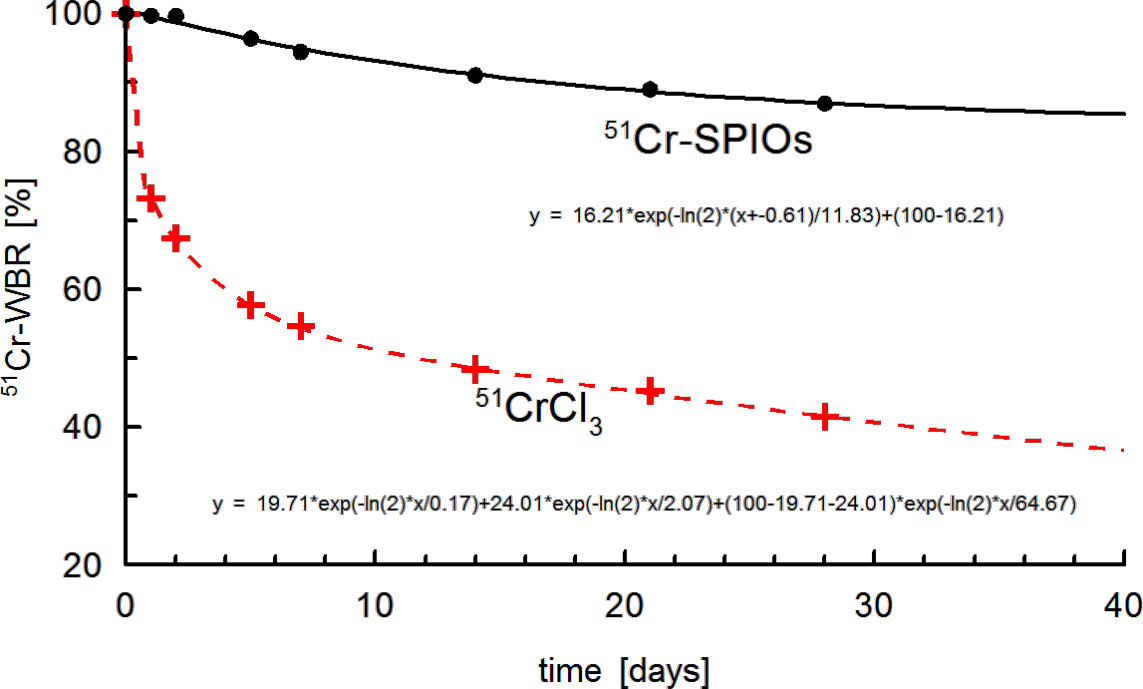
Whole body retention (WBR) of ^51^Cr-SPIOs and ionic chromium after intravenous injection. The fitted curve of ^51^CrCl_3_ follows a standard triple exponential decay curve for a 3-compartment model (transport, transit, storage pool, with half-life 0.2, 2.1, ≈65 d).

The whole body retention (WBR) curve shown in Figure 4 for ^51^Cr-SPIOs clearly shows a lag phase of about 2 d, in which ^51^Cr was excreted from the whole body. Using a correction term, a 2-compartment model was used for fitting which resulted in a relatively short half-life of 12 d, and very long half-life of ≈1000 d, which cannot be derived precisely from the fit.

As a result, the ^51^Cr label seems adequate for labeling the nanoparticles during blood passage and cell uptake. However, as soon as intracellular degradation of the core takes place, the different transport paths for Fe^2+^/Fe^3+^ and Cr^3+^ become a consideration. Iron is dissolved from the iron oxide cores within lysosomes under acid conditions, which then further reduces to Fe^2+^ by Steap3 and is transported across the endosomal membrane by the divalent metal ion transporter, DMT1 [26]. During iron homeostasis, excess iron would then be released by, for example, macrophages via ferroportin***,*** bound to apotransferrin and transported to cells in need for iron. The intracellular processing of Cr^3+^ is more or less unknown, and even its physiological role as a trace element is controversially discussed. Supporters of its physiological role have suggested a function in the form of a Cr^3+^-binding peptide, the so-called chromodulin, occurring in carbohydrate, fat, or cholesterol, whereby metabolism can increase insulin sensitivity in insulin-dependent cells [27]. In this model, after interaction, Cr^3+^ would be excreted via the kidneys and replaced by freshly absorbed Cr [28]. As shown here by the ^51^Cr-WBR over 28 d, the excretion of ^51^Cr via the kidneys and gastrointestinal tract is rather slow when using ^51^Cr-SPIOs and very different from ^51^CrCl_3_ (Figure 4). Excretion of ^51^Cr from SPIOs was found mostly in feces and very little in urine (data not shown). After 4 weeks, a small loss of ^51^Cr from the liver seems to be distributed mainly to the spleen, gastrointestinal tract (GIT), and carcass, whereas from CrCl_3_, ^51^Cr accumulates in the liver. These results indicate a striking different metabolism of Cr^3+^ from these two sources. We know from previous experiments with the same SPIOs labeled with ^59^Fe instead of ^51^Cr that the iron oxide core of this particle is degraded to a large extent in the liver (data not shown). We therefore speculate here that the intracellular release of ^51^Cr is trapped within the liver cells due to the lack of a specific Me^3+^ exporter.

**Figure 5:**
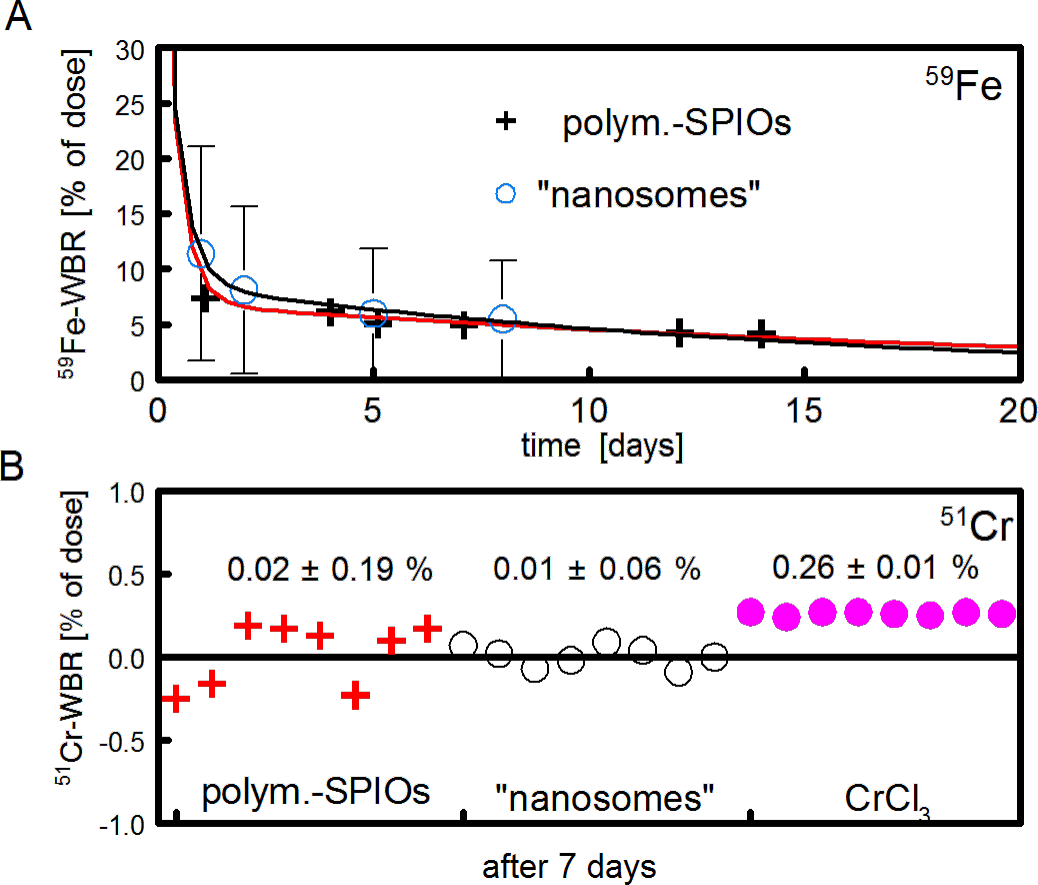
Absorption of ^59^Fe- or ^51^Cr-labeled SPIOs in mice. (A) ^59^Fe-labeled polymer-coated SPIOs or so-called “nanosomes” (oleic acid-stabilized, hydrophobic SPIOs embedded in chylomicron-like lipid micelles) [29] were administered by gavage to groups of mice (*n* = 4–5). The ^59^Fe-WBR was measured after 1–14 d. (B) The same procedure as with ^51^Cr-labeled SPIOs, “nanosomes” and ^51^CrCl_3_ (*n* = 8). Values of the respective ^51^Cr-WBR for each individual mouse is given at day 7 day (which is taken as the apparent gastrointestinal ^51^Cr absorption).

Thus, our results could be interpreted as a new argument against the role of Cr^3+^ as a physiological trace element. From this perspective, the ^51^Cr labeling of SPIOs would present a novel way to bring ^51^Cr into cells and to study possible transport paths of this element, however, it seems less appropriate to study the fate of SPIOs in vivo. However, we present here an interesting application in which the differences between the metabolism of iron and chromium can be elegantly used to study the absorption of intact nanoparticles from the gastrointestinal tract, an important topic in nanotoxicology. For this, ^59^Fe-labeled SPIOs were given by gavage to groups of mice and the ^59^Fe-WBR was followed in living mice for 14 d using a whole body counter (Figure 5A). Most of the activity was lost via fecal excretion within the first 3 d, which is typical for rodents. The ^59^Fe-WBR at days 7–10, a well-accepted parameter for the intestinal iron absorption, showed a small but significant absorption rate of about 5% of the dose (Figure 5A). The problem is that substantial amounts of ionic iron can also be absorbed in the duodenum by the physiological absorption mechanism via DMT1. Therefore, the ^59^Fe-results can simply be interpreted as a partial digestion of the SPIOs in the stomach followed by the absorption of released ionic Fe^2+^ and do not necessarily show the absorption of intact nanoparticles.

The use of ^51^Cr-labeled SPIOs can clarify this point, because it was shown earlier that the intestinal absorption of ionic Cr^3+^ is extremely low in rodents [25,30]. When ^51^Cr-labeled SPIOs were orally administered to groups of mice, no absorbed activities were detected from polymer-coated SPIOs or from oleic acid-stabilized, lipophilic SPIOs embedded in lipid micelles (Figure 5B, “nanosomes”). The measured ^51^Cr values were significantly lower (*p* < 0.01) as compared to a trace dose of orally administered, aqueous CrCl_3,_ indicating that only a very limited amount (<0.05%) of the administered dose from intact particles can be absorbed in the intestinal tract. This excludes a relevant, unspecific particle uptake in the intestinal tract, at least for particles of this type (size, charge).

It should be noted that the results obtained with lipid micelles are also relevant for the field of dietary fat absorption in the intestinal tract, and would support the classical view of fatty acid absorption from micelles formed in the gastrointestinal tract after food intake. The action of bile acids and pancreas lipase would first produce free fatty acids or monoglycerides, but obviously does not include the absorption of intact micelles into enterocytes [31].

### ^65^Zn-labeling of quantum dots

For intrinsic labeling, a variety of radionuclides can be incorporated into quantum dots consisting of cadmium, copper, indium, zinc, selenium, and tellurium. Although cadmium is the most relevant, it has no adequate radioisotope that would allow for easy availability and optimal detection for in vivo studies. Therefore, studies with ^109^Cd are rare. However, in a recent proof of principle study, this radionuclide was used to synthesize CdTeSe/^109^CdZnSe and to study the distribution in mice up to 7 d [32].

**Figure 6:**
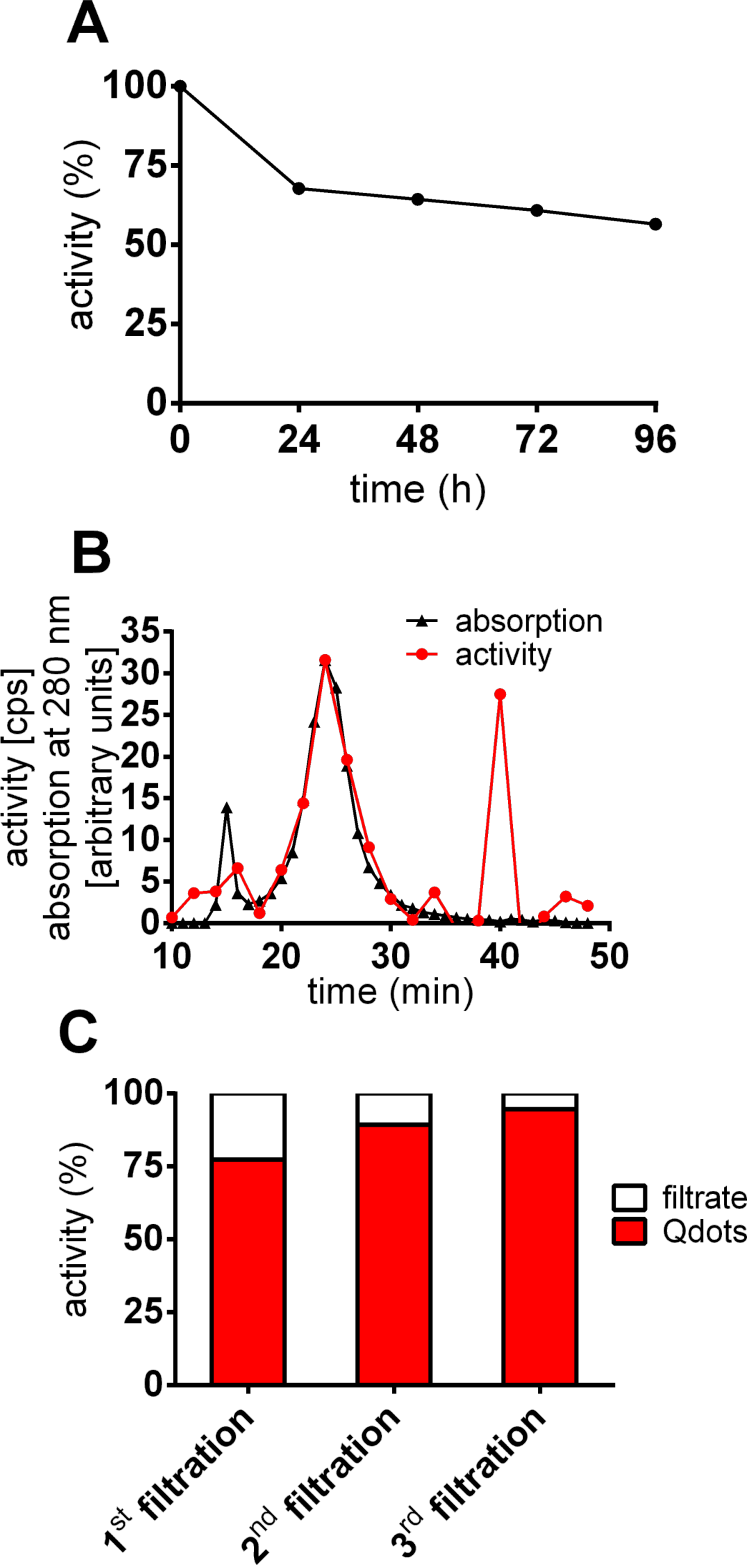
Stability of ^65^Zn-radiolabeled Qdots with a polymer coating. (A) Dialysis of ^65^Zn-radiolabeled and polymer-coated Qdots. The Qdots were transferred to a dialysis tube and the dialysis buffer was removed every 24h and tube and buffer were measured. ^65^Zn appears not to be stably incorporated: there is small release (≈4%/24h) of ^65^Zn^2+^ ions. (B) SEC of ^65^Zn-radiolabeled and polymer-coated Qdots. (C) Desalting of Qdots in a 10 kDa centrifugal filter unit. The radioactivity in the filtrate indicates a slight loss of the radioactive label. The resuspension and filtration of the Qdots was repeated two times. The black line shows the elution profile of the nanoparticles whereas the red line represents the profile of the activity of ^65^Zn. Nanoparticles and radioactivity are located in the same fraction (24 min), although unbound ^65^Zn ions can also be found (40 min) directly after filtration.

In the present study the outer ZnS shell of CdSe/CdS/ZnS (core/shell/shell) quantum dots was labeled by incubation with water-free ^65^Zn^2+^ in an organic solvent. This radionuclide is a hard γ-emitter (1.1 MeV), which would allow precise measurement even in living animals using a whole body counter. Precipitation with methanol revealed that over 70% of the radioactivity was localized within the Qdot-mass, which hints at the accumulation of the isotope in the outer shell. Stability tests of the polymer-coated Qdots included dialysis, size-exclusion chromatography (SEC) and filtration techniques. Dialysis took place against phosphate buffered saline (PBS) and the buffer was changed every 24 h (Figure 6A). Around 30% of the radioactivity was found in the dialysate after 24 h. Only around 4% of the radioactivity was detected in the removed buffer after 48 h. Similar results were obtained for samples taken at 72 h and 96 h. The amount of radioactivity that was released into the buffer after the first 24 h could be attributed to free ^65^Zn ions from the encapsulation technique. Further loss of the label during the course of time could indicate a small but continuous bleeding of the radioactive isotope. These results were confirmed by filtration using a centrifuge to concentrate the probes (Figure 6B).

Radiolabeled Qdots were filtered and thereby separated from free ^65^zinc ions. The concentrated Qdots were resuspended and the filtration procedure was repeated twice. 22.6, 10.6 and 5.4% of the radioactivity was found in the filtrate after the first, second and third filtration. The SEC of the radiolabeled, hydrophilic Qdots shows that the particles basically elute after 24 min (Figure 6C). It can be clearly seen that most of radioactivity is colocalized with the nanoparticles. Nevertheless, there was also a fraction of ionic ^65^Zn that eluted after 40 min and cannot be completely separated by centrifugal filtration due to the slight continuous bleeding of the label (or the dissolution of the ZnS shell).

### Pharmacokinetic measurements of ^65^Zn-labeled Qdots

**Figure 7:**
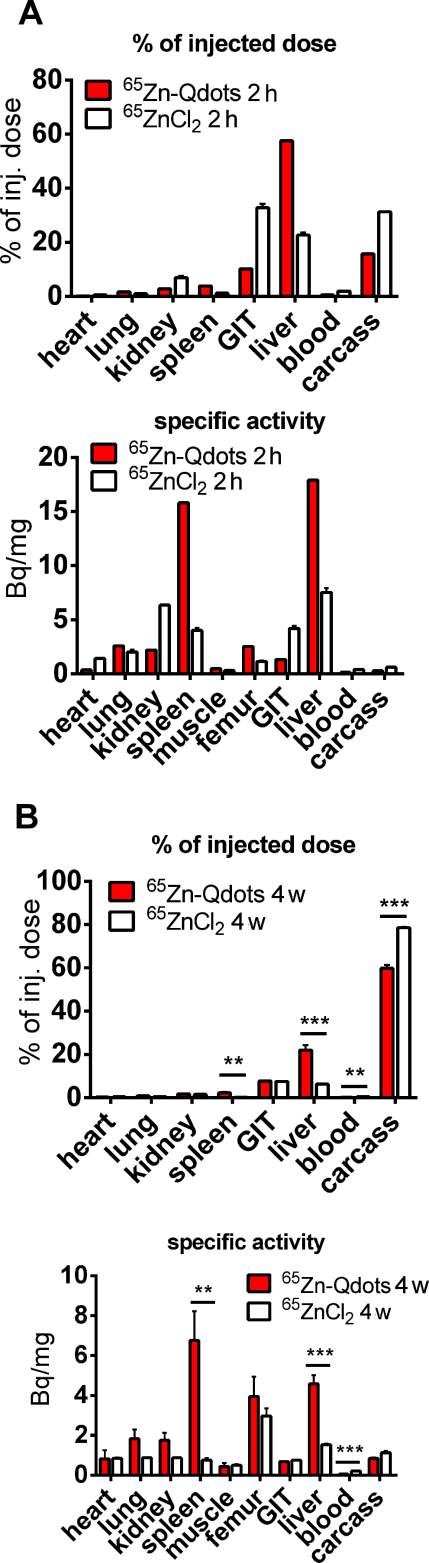
Organ distribution of Qdots and ionic zinc after intravenous injection into the tail vein of mice. ^65^Zn activity was measured in organs and tissue. The percentage distribution of polymer-coated Qdots and ionic zinc 2 h (A) and 4 weeks (4 w) (B) after application is shown. The liver and spleen have the highest specific activity when Qdots are applied. The data are presented as mean values ± standard error of the mean.

Despite the described stability problems of the ^65^Zn-label, the radiolabeled Qdots were used to investigate basic pharmacokinetic properties under these conditions. Zinc is a well-known trace element and widely used as a catalytic or structural cofactor in about 3000 human zinc proteins [33–34]. At least 14 speciﬁc transporters are responsible for either zinc inﬂux or efﬂux in mammalian cells [35]. Detailed compartment models of zinc kinetics in humans and mice are available [36–37]. As cadmium and zinc both belong to the group IIB of the periodic table, they form tetrahedral complexes and compete for the same binding sites and/or ligands in biological systems [38]. Their interaction is probably one of the most recognized metal–metal interactions, which could make ^65^Zn as acceptable marker also for Cd metabolism. The organ distribution 2 h after administration shows the liver and the spleen are the major uptake organs for Qdots with around 70% of the injected dose present (Figure 7A). This is in good agreement with the results from Sun et al. [32] and supports the reliability of our label results.

**Figure 8:**
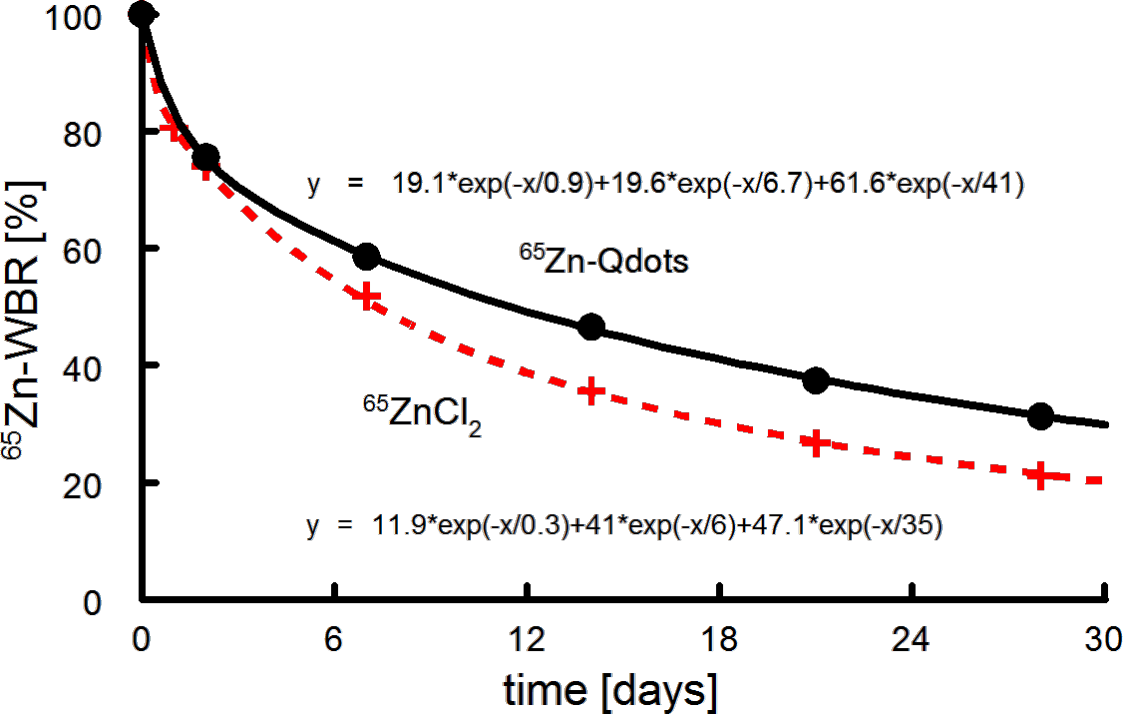
Whole body retention of Qdots and ionic zinc in mice (*n* = 4). Curves indicate fits using a triple exponential decay function for a 3-compartment model. Similar half-lives with different fractions were found for Qdots (0.9, 6, 41 d, with fractions 19.1, 19.6, and 41%) and ZnCl_2_ (0.3, 6.7, 35 d, 11.9, 41, and 35%). Nevertheless, the difference between the curves is significant (*p* < 0.05), indicating an incomplete degradation of particles in the liver.

When CdSe/CdS/ZnS (core/shell/shell) quantum dots are degraded in vivo, the outer shell will be dissolved first and zinc will be released into the system. The Qdot distribution in organs 2 h and 4 weeks after intravenous injection was investigated. To compare the data to a physiological distribution of zinc, ^65^ZnCl_2_ was used as a control against ^65^Zn-radiolabeled Qdots. As discussed earlier, this is a necessary control for studying the fate of any given degradable particle. In an autoradiographic study, the highest uptakes after 2 h were found in the liver, pancreas, spleen, kidneys and the intestinal walls, which is in good agreement with our values for ZnCl_2_ shown in Figure 4 [39]. However, also in short-time experiments, the liver is not the main storage organ for ionic Zn and as became obvious from the short-time distribution of the ^65^Zn-label between ^65^ZnCl_2_ and ^65^Zn-Qdots. Zn has a fast turnover, and the majority of the ^65^Zn label is removed after 4 weeks from all organs that have fast exchange kinetics and remains stored in tissue with low exchanges rates (such as bones and skin). Organ distribution of ^65^Zn-labeled Qdots and a trace ^65^Zn dose 4 weeks after intravenous injection was found to be similar for most of the organs and tissue, with the exception of the liver and spleen. This indicates that radioactivity in the form of Qdots is stored in these organs, and the particles are thus not fully degraded. Otherwise, ^65^Zn released by degradation of the nanocrystals was distributed between all organs in a manner comparable to that of the ionic ^65^ZnCl_2_. In contrast to the chromium results discussed above, ^65^Zn from CdSe/CdS/ZnS-Qdots shows a similar whole body retention compared with ^65^ZnCl_2_ (Figure 8).

### Distribution of Qdots within the liver

**Figure 9:**
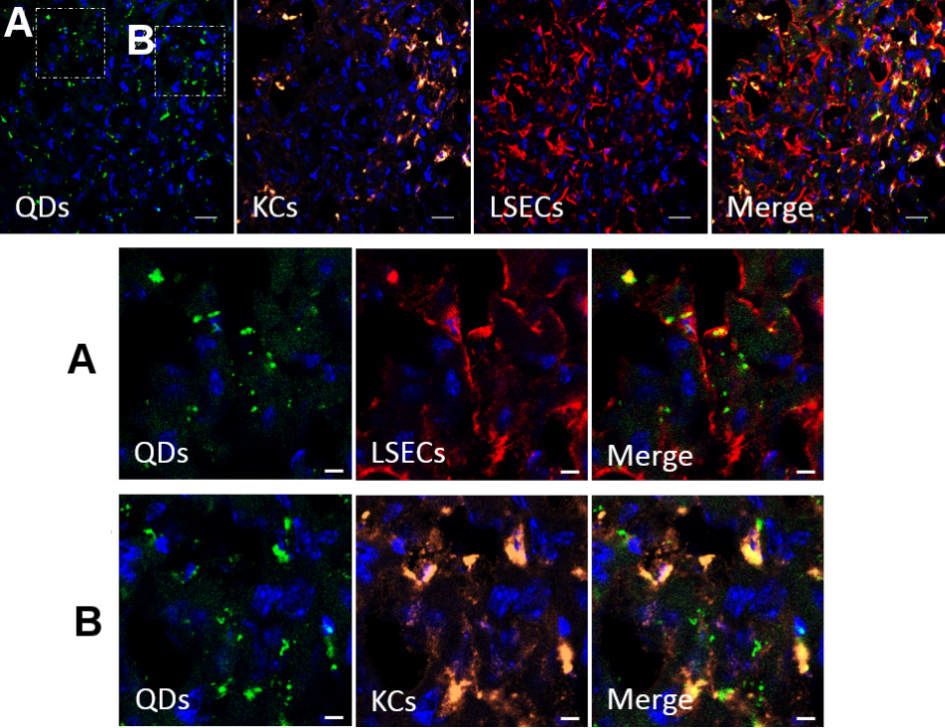
Confocal microscopy of a cryosection of a rat liver 2h after intravenous injection of polymer-coated Qdots. The nuclei are stained with DAPI. Immunostaining of Kupffer cells (KCs, anti-CD31) and liver sinusoidal endothelial cells (LSECs, with anti-CD68) was performed. Regions outlined by the white boxes are magnified in the lower panels. Nanoparticles can be found located in endothelial cells (A) as well as in Kupffer cells (B), but not in hepatocytes. Scale bar: 20 µm for the upper panel, and 5 µm for the magnified images.

Since the radiolabeled Qdots show that the liver is the major uptake organ of the analyzed nanomaterials, a closer look at the distribution of Qdots among different liver cells was warranted. Therefore, polymer-coated Qdots were injected and the mice were observed two hours after intravenous injection into the tail vein and cryosections of the liver were prepared (Figure 9).

It is well-established that liver sinusoidal endothelial cells (LSECs) carry out a scavenger function by expressing several types of scavenger receptors and that Kupffer cells (KCs) belong to the family of macrophages and form part of the reticuloendothelial system. Thus, the sections were analyzed by immunofluorescence and stained for hepatic endothelial cells and Kupffer cells, which are known to play important roles in endocytosing processes and are known to be among the most prominent cell types which take up protein-corona-covered nanoparticles [22,40]. The confocal images of the cryosections exhibit the cell distribution within the liver and confirm this hypothesis. Internalized Qdots colocalize with LSECs as well as with KCs. To date, polymer-coated Qdots were not found in hepatocytes. Since the surface chemistry of the Qdots and SPIOs is identical when coated with the amphiphilic polymer, the cell distribution should be similar.

### Intracellular processing of Qdots

**Figure 10:**
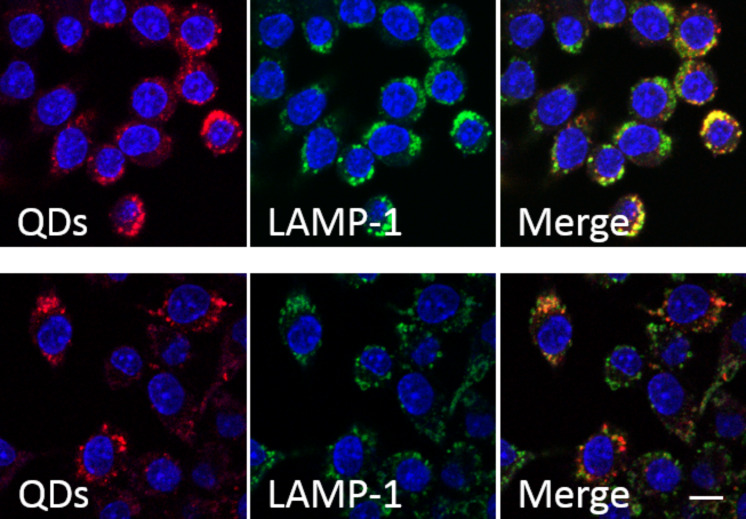
Colocalization of Qdots and lysosomes. J774 cells were incubated with Qdots (red) for 2 h and fixed afterwards (upper row) or 24 h later (lower row). Cells were immunostained with anti-LAMP1 (detected with Cy2, green). The “Merge” images show that Qdots are localized in lysosomes (yellow). Scale bar: 50 µm.

Further insight into the cellular internalization and processing of polymer-coated Qdots and SPIOs was gained by cell culture. For comparison of the in vivo data, these studies were made with J774 cells (a murine macrophage cell line). The cells were incubated with polymer-coated Qdots for 2 h, then the nanoparticles were removed, and the cells were rested for 24 h. The cells were checked for degradation, which would be indicated by decreasing fluorescence over time due to cell division (Figure 10).

When cells were fixed directly after incubation, the red fluorescence of the Qdots was found to colocalize with the green fluorescence from lysosomes labeled with the LAMP1 antibody, which indicates that the nanoparticles accumulate in the lysosomes. When the cells were fixed 24 h later, the Qdots fluorescence still colocalizes with lysosomes and does not show evidence of degradation, meaning that the possible degradation process seems too slow to be followed by a change in fluorescence.

However, in a group of mice, the liver cryosections were repeated 12 weeks after intravenous injection. Here no further localizable fluorescence was observed, indicating that most of the Qdots in the liver were at least partially degraded, resulting in a loss of fluorescence. This would otherwise confirm the ^65^Zn results, however, this was in contrast to Yank et al. who followed commercial PEG 5000-coated CdTe/ZnS Qdots by tracing their fluorescence in mice for 28 d and measured ^111^Cd by inductively coupled plasma–mass spectrometry (ICP–MS) [41]. In this study, they found high levels of cadmium in the spleen, liver, and kidneys. Additionally, Su et al. found persistent, initial, high cadmium concentrations in the liver and spleen, which remained at high levels even after 80 d [42]. However, in both these studies, the cadmium concentrations in organs cannot directly be substituted for intact Qdots, and differences in Cd concentrations between organs would not necessarily indicate a redistribution of particles in vivo. So far, to our knowledge, the export of nanoparticles from living cells may occur as single particles crossing barriers, but this has never been directly observed for a flux of particles.

## Conclusion

The present study highlights the importance of reliable quantification methods for in vivo studies involving quantum dots. Radiolabeling could provide such quantification, preferentially by introduction of an appropriate radionuclide into the core or an inner shell during chemical synthesis in order to guarantee the full stability of the label.

## Experimental

### Nanoparticle synthesis

Monodisperse, oleic acid-stabilized SPIOs were synthesized according to Yu et al. [43]. Briefly, a mixture of 2 mmol FeOOH, 8.0 mmol oleic acid and 22 mL of 1-octadecene was held at 320 °C under nitrogen for about 80 min. The 11 nm core diameter particles showed a narrow size distribution (less than 10% standard deviation) as confirmed by transmission electron microscopy (TEM). Encapsulation with the PMAOD solution was achieved using a sonification method as described in [24]. From the measured iron content, the diameter of the core (11 nm) and the assumed core material (Fe_2_O_3_), the molar concentration of an aqueous solution was calculated.

CdSe/CdS/ZnS (core/shell/shell) quantum dots with red (7 nm diameter) or green (5.5 nm) fluorescence were synthesized as described in [29]. This was done as a one-pot synthesis using a mixture of hexadecylamine (HDA), trioctylphosphine oxide (TOPO), and trioctylphosphine (TOP) as a stabilizing solvent, following the techniques described by Mekis [44]. As the core size of Qdots is directly related to the excitonic peak in the UV–vis absorption spectrum, the size-dependent molar extinction coefficient for the CdSe core was determined using the following empirical formula [45]: D = (1.6122∙10^−9^)λ^4^ − (2.6575∙10^−6^)λ^3^ + (1. 6242∙10^−3^)λ^2^ − (0.4277)λ + 41.57.

### Radioactive labeling SPIOs and Qdots

The radionuclides were purchased from Perkin-Elmer, Rodgau, Germany (^51^CrCl_3_ in 0.5 HCl, specific activity: 700 mCi/mg; ^65^ZnCl_2_ in 0.5 N HCl, specific activity: 4.32 mCi/mg).

For the radiolabeling of SPIOs, similar to that previously described [24], aliquots of ^51^CrCl_3_ (40–80 µCi, 0.3–0.6 µg Cr) were lyophilized to remove water and traces of hydrochloric acid. Next, previously synthesized, monodisperse, oleic acid-stabilized SPIOs were added (1 mg dry weight in 400 µL CHCl_3_). The solution was stirred at room temperature for at least 24 h before using the SPIOs for further experiments (i.e., embedding in lipid micelles or polymer coating).

For the synthesis of the ^65^Zn-Qdots, an aliquot of an acidic ^65^ZnCl_2_ solution (20–40 µCi, 13–25 µg Zn) was lyophilized to dryness, and a solution of lyophilic Qdots in chloroform was added and stirred at room temperature for 24 h. The same polymer as described above was used for the transfer into aqueous solution.

As control solutions, 100 µL of aqueous ^51^CrCl_3_ (per mouse 1.5–3.0 µCi, 14 ng Cr) or aqueous ^65^ZnCl_2_ (per mouse 2–2.6 µCi, 1.3–1.8 µg Zn) was intravenously injected. For the animal experiments, the applied Cr content in the injected volumes was completely negligible as compared to the natural concentration of Cr(III) found in a mouse. The specific activity of the ^65^Zn solutions available were already rather low, however, the applied nominal Zn content/per mouse (0.2–1.8 µg Zn) was also well below the daily flux of Zn within a mouse from the uptake due to a normal diet of 260 µg Zn [35–37].

### Size-exclusion chromatography (SEC)

Similar to that previously described in [24], SEC was performed using a Superose-6 10/300 GL column (Amersham Bioscience, Munich, Germany) with buffer (10 mM tris(hydroxymethyl)aminomethane, 0.15 mM NaCl, 10 mM EDTA) at a flow rate of 0.5 mL/min. Particle detection was achieved via UV absorption at 280 nm.

For iron detection, 200 µL of each fraction was treated with 50 µL of 5 M hydrochloric acid at 70 °C for 30 min. Afterwards, 150 µL of a 2 M acetate buffer (pH 4.8) containing 10% ascorbic acid was added to 50 µL of each fraction, followed by 100 µL of a solution of 50 mg bathophenanthroline in 50 mL water. After 15 min, the absorption was measured at 540 nm.

### Radioactivity measurements

^51^Cr- and ^65^Zn-activity in living mice or tissue samples were measured using the Hamburg whole body radioactivity counter. The whole body retention of ^51^Cr or ^65^Zn was measured at given time points in a 200 cm long, 4π geometry, whole body radioactivity detector with a liquid organic scintillator in the energy range from 980–3,000 keV [46]. The respective mouse was placed in a cage in the middle of the counter. The influence of the movement of the animal during the measurement (1–6 min) was negligible due to the large dimension of the counter. After intravenous application, repeated measurements of the ^51^Cr or ^65^Zn activity were performed over the course of 28 d (Figure 4 and Figure 8) and are a quantitative measure of the excretion of the specific isotope through feces and urine. After oral administration, the whole body retention after 7–14 d is a well-accepted measure of the intestinal absorption of the isotopes in the respective galenic formulation, because non-absorbed material is completely excreted at that time via the feces.

### In vivo studies

All animal experiments were approved by the local committee for animal experiments (Behörde für Soziales, Familie, Gesundheit und Verbraucherschutz, BSG, Hamburg Tierversuchs-Nr. 34/10).

Wild type FVB/N mice were injected with 100–200 µL of a solution containing either ^51^Cr-SPIOs (per mouse: 50–100 µg, Fe = 25–50 pmol particles, molar ratio ^51^Cr:Fe = 1:1700–3400) or ^65^Zn-Qdots (per mouse: 50–100 pmol, with 0.1–0.3 µg Zn and 0.2–0.35 µCi ^65^Zn) into the tail vein. The activity, measured immediately after administration of the radiolabeled compounds in the whole body, was taken as the 100% reference value. At 120 min or 4 weeks after injection, the mice were anaesthetized (Rompun/Ketamine), blood was removed by cardiac puncture, and the organism was perfused with PBS containing 10 units of heparin. The blood and the organs (spleen, kidney, liver, etc.) were removed and weighed and the radioactivity was measured.

To prepare cryosections of the liver, the mice were anaesthetized after 2 h after administration of Qdots, and the organism was perfused with PBS containing 4% PFA and 5% sucrose. The liver was removed and allowed to further rest for 60 min on ice. Tissue sections of 10 µm thicknesses were cut from frozen specimens embedded in an optimum cutting temperature formulation solution (Sakura Finetek Europe). Cryosections were mounted on a slide (Superfrost/Plus, Glaswarenfabrik Karl Hecht KG, Sondheim, Germany) and dried overnight before immunostaining.

### Cell culture

J774 cells were cultured on microscope cover glasses (13 mm diameter, Glaswarenfabrik Karl Hecht KG, Sondheim, Germany) in DMEM medium (10% FCS, 4.5 g/L glucose, [+]glutamine, [−]pyruvate, 1% antimycotic–antibiotic from Invitrogen). The cells were incubated at 37 °C in a humidified atmosphere containing 5% CO_2_. For uptake analysis, the J774 cells were seeded onto 24-well plates at a density of 100,000 cells/well and allowed to grow for 2 d. Typically, the cells were incubated with 500 µL of medium containing Qdots (8.4 nM) at 37 °C. After 2 h, the medium was removed from each well and fixed with 4% PFA or the cells were allowed to rest for another 24 h before fixing for immunofluorescence studies.

### Fluorescent immunostaining

For immunostaining, the cryosection slides were blocked with 1% BSA in PGS (PBS, 0.5 mg/mL saponin, 5 mg/mL glycine) for 1 h at room temperature. After blocking, the slides were incubated with the respective primary antibodies at 4 °C overnight. Afterwards, the slides were washed three times with PGS followed by incubation with secondary antibodies for 2 h at room temperature.

For in vitro immunofluorescence studies, cells were likewise blocked with 1% BSA in PGS for 1 h at room temperature, followed by incubation with primary antibody for 1 h at 37 °C and three washes with PGS. Incubation with secondary antibody was performed for 45 min at 37 °C.

The following antibodies and dilutions were used: rabbit anti-CD31 (1:50, Abcam), mouse anti-Lamp1 (1:200, Developmental Studies Hybridoma Bank) and rat anti-CD68 (1:100, Abcam); Cy3 anti-rabbit (1:100, Jackson Immuno Research Laboratories), Alexa 488 anti-mouse (1:250, Jackson Immuno Research Laboratories) and Alexa 647 anti-rat (1:50; Jackson Immuno Research Laboratories). Nuclei were stained with DAPI.

### Data analysis

To assess the statistical significance between results in different groups of mice, the two-tailed, unpaired, Student’s *t*-test was performed, where the parameter P < 0.05 was considered as significant.

Slide Write Plus 7.0 software (Advanced Graphics Software Inc., Encinitas, USA) was used to fit the data for the whole body retention in mice (Figure 4 and Figure 8).

The mean values of the ^51^Cr or ^65^Zn retention data *R*(*t*) from whole body counting were fitted by a 3-compartment model

[1]
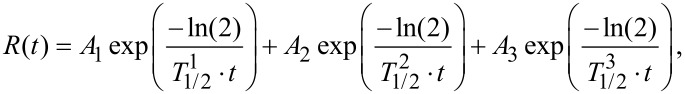


where the fractions *A*_1_ + *A*_2_ + *A*_3_ = 100% and the corresponding half-lives are 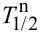.
